# Supplementary material for: Unveiling the Bioactive Potential of the Invasive Jellyfish Phyllorhiza punctata Through Integrative Transcriptomic and Proteomic Analyses
Source: Biomolecules. 2025 Aug 4;15(8):1121. doi: 10.3390/biom15081121 (PMC12383608; doi:10.3390/biom15081121)
Supplement: Supplementary file 1 [file biomolecules-15-01121-s001.zip › Suplementary Material/FileS3/PEPFOLD4/XP_065058821_11728/clusters.html]

 PEP-FOLD clusters


Download archive of all models  here   

|  |  |  |
| --- | --- | --- |
| # | file | sOPEP |
| # cluster 1 - Size: 54 |  |  |
|  | XP\_065058821\_11728-model1 | -12.6985 |
|  | XP\_065058821\_11728-model1.1 | -12.6219 |
|  | XP\_065058821\_11728-model1.2 | -12.6088 |
|  | XP\_065058821\_11728-model1.3 | -12.6088 |
|  | XP\_065058821\_11728-model1.4 | -12.5771 |
|  | XP\_065058821\_11728-model1.5 | -12.5771 |
|  | XP\_065058821\_11728-model1.6 | -12.5442 |
|  | XP\_065058821\_11728-model1.7 | -12.5442 |
|  | XP\_065058821\_11728-model1.8 | -12.5333 |
|  | XP\_065058821\_11728-model1.9 | -12.5266 |
|  | XP\_065058821\_11728-model1.10 | -12.5266 |
|  | XP\_065058821\_11728-model1.11 | -12.5102 |
|  | XP\_065058821\_11728-model1.12 | -12.503 |
|  | XP\_065058821\_11728-model1.13 | -12.503 |
|  | XP\_065058821\_11728-model1.14 | -12.4645 |
|  | XP\_065058821\_11728-model1.15 | -12.4645 |
|  | XP\_065058821\_11728-model1.16 | -12.4635 |
|  | XP\_065058821\_11728-model1.17 | -12.4607 |
|  | XP\_065058821\_11728-model1.18 | -12.4607 |
|  | XP\_065058821\_11728-model1.19 | -12.4607 |
|  | XP\_065058821\_11728-model1.20 | -12.4607 |
|  | XP\_065058821\_11728-model1.21 | -12.4554 |
|  | XP\_065058821\_11728-model1.22 | -12.4485 |
|  | XP\_065058821\_11728-model1.23 | -12.4485 |
|  | XP\_065058821\_11728-model1.24 | -12.4485 |
|  | XP\_065058821\_11728-model1.25 | -12.4485 |
|  | XP\_065058821\_11728-model1.26 | -12.4485 |
|  | XP\_065058821\_11728-model1.27 | -12.4485 |
|  | XP\_065058821\_11728-model1.28 | -12.4308 |
|  | XP\_065058821\_11728-model1.29 | -12.4271 |
|  | XP\_065058821\_11728-model1.30 | -12.4063 |
|  | XP\_065058821\_11728-model1.31 | -12.4063 |
|  | XP\_065058821\_11728-model1.32 | -12.4022 |
|  | XP\_065058821\_11728-model1.33 | -12.3693 |
|  | XP\_065058821\_11728-model1.34 | -12.3681 |
|  | XP\_065058821\_11728-model1.35 | -12.3526 |
|  | XP\_065058821\_11728-model1.36 | -12.344 |
|  | XP\_065058821\_11728-model1.37 | -12.3209 |
|  | XP\_065058821\_11728-model1.38 | -12.2924 |
|  | XP\_065058821\_11728-model1.39 | -12.2874 |
|  | XP\_065058821\_11728-model1.40 | -12.2847 |
|  | XP\_065058821\_11728-model1.41 | -12.2825 |
|  | XP\_065058821\_11728-model1.42 | -12.2797 |
|  | XP\_065058821\_11728-model1.43 | -12.2767 |
|  | XP\_065058821\_11728-model1.44 | -12.2748 |
|  | XP\_065058821\_11728-model1.45 | -12.2738 |
|  | XP\_065058821\_11728-model1.46 | -12.2571 |
|  | XP\_065058821\_11728-model1.47 | -12.2414 |
|  | XP\_065058821\_11728-model1.48 | -12.1033 |
|  | XP\_065058821\_11728-model1.49 | -12.0119 |
|  | XP\_065058821\_11728-model1.50 | -11.9678 |
|  | XP\_065058821\_11728-model1.51 | -11.9381 |
|  | XP\_065058821\_11728-model1.52 | -11.7623 |
|  | XP\_065058821\_11728-model1.53 | -11.6706 |
| # cluster 2 - Size: 32 |  |  |
|  | XP\_065058821\_11728-model2 | -12.1951 |
|  | XP\_065058821\_11728-model2.1 | -12.1313 |
|  | XP\_065058821\_11728-model2.2 | -12.0674 |
|  | XP\_065058821\_11728-model2.3 | -12.0354 |
|  | XP\_065058821\_11728-model2.4 | -12.0178 |
|  | XP\_065058821\_11728-model2.5 | -11.9682 |
|  | XP\_065058821\_11728-model2.6 | -11.9682 |
|  | XP\_065058821\_11728-model2.7 | -11.9538 |
|  | XP\_065058821\_11728-model2.8 | -11.9538 |
|  | XP\_065058821\_11728-model2.9 | -11.9538 |
|  | XP\_065058821\_11728-model2.10 | -11.9538 |
|  | XP\_065058821\_11728-model2.11 | -11.9236 |
|  | XP\_065058821\_11728-model2.12 | -11.8979 |
|  | XP\_065058821\_11728-model2.13 | -11.8916 |
|  | XP\_065058821\_11728-model2.14 | -11.8857 |
|  | XP\_065058821\_11728-model2.15 | -11.865 |
|  | XP\_065058821\_11728-model2.16 | -11.8399 |
|  | XP\_065058821\_11728-model2.17 | -11.8398 |
|  | XP\_065058821\_11728-model2.18 | -11.8207 |
|  | XP\_065058821\_11728-model2.19 | -11.8164 |
|  | XP\_065058821\_11728-model2.20 | -11.7916 |
|  | XP\_065058821\_11728-model2.21 | -11.7738 |
|  | XP\_065058821\_11728-model2.22 | -11.7738 |
|  | XP\_065058821\_11728-model2.23 | -11.7654 |
|  | XP\_065058821\_11728-model2.24 | -11.7331 |
|  | XP\_065058821\_11728-model2.25 | -11.716 |
|  | XP\_065058821\_11728-model2.26 | -11.7107 |
|  | XP\_065058821\_11728-model2.27 | -11.6822 |
|  | XP\_065058821\_11728-model2.28 | -11.6777 |
|  | XP\_065058821\_11728-model2.29 | -11.6685 |
|  | XP\_065058821\_11728-model2.30 | -11.5948 |
|  | XP\_065058821\_11728-model2.31 | -11.4024 |
| # cluster 3 - Size: 5 |  |  |
|  | XP\_065058821\_11728-model3 | -11.9437 |
|  | XP\_065058821\_11728-model3.1 | -11.8367 |
|  | XP\_065058821\_11728-model3.2 | -11.701 |
|  | XP\_065058821\_11728-model3.3 | -11.6189 |
|  | XP\_065058821\_11728-model3.4 | -11.5739 |
| # cluster 4 - Size: 3 |  |  |
|  | XP\_065058821\_11728-model4 | -11.9 |
|  | XP\_065058821\_11728-model4.1 | -11.7998 |
|  | XP\_065058821\_11728-model4.2 | -11.7792 |
| # cluster 5 - Size: 6 |  |  |
|  | XP\_065058821\_11728-model5 | -11.8677 |
|  | XP\_065058821\_11728-model5.1 | -11.7562 |
|  | XP\_065058821\_11728-model5.2 | -11.7351 |
|  | XP\_065058821\_11728-model5.3 | -11.657 |
|  | XP\_065058821\_11728-model5.4 | -11.6413 |
|  | XP\_065058821\_11728-model5.5 | -11.5706 |
|  |  |  |
